# The Allocation of Valenced Percepts Onto 3D Space

**DOI:** 10.3389/fpsyg.2019.00352

**Published:** 2019-02-27

**Authors:** Fernando Marmolejo-Ramos, Artin Arshamian, Carlos Tirado, Raydonal Ospina, Maria Larsson

**Affiliations:** ^1^School of Psychology, The University of Adelaide, Adelaide, SA, Australia; ^2^Gösta Ekman Laboratory, Department of Psychology, Stockholm University, Stockholm, Sweden; ^3^CAST Laboratory, Department of Statistics, Federal University of Pernambuco, Recife, Brazil

**Keywords:** embodied cognition, metaphorical mapping, valence-space metaphor, cognition cube, approach-avoidance

## Abstract

Research on the metaphorical mapping of valenced concepts onto space indicates that positive, neutral, and negative concepts are mapped onto upward, midward, and downward locations, respectively. More recently, this type of research has been tested for the very first time in 3D physical space. The findings corroborate the mapping of valenced concepts onto the vertical space as described above but further show that positive and negative concepts are placed close to and away from the body; neutral concepts are placed midway. The current study aimed at investigating whether valenced perceptual stimuli are positioned onto 3D space akin to the way valenced concepts are positioned. By using a unique device known as the cognition cube, participants placed visual, auditory, tactile and olfactory stimuli on 3D space. The results mimicked the placing of valenced concepts onto 3D space; i.e., positive percepts were placed in upward and close-to-the-body locations and negative percepts were placed in downward and away-from-the-body locations; neutral percepts were placed midway. These pattern of results was more pronounced in the case of visual stimuli, followed by auditory, tactile, and olfactory stimuli.

Significance Statement

Just recently, a unique device called “the cognition cube” (CC) enabled to find that positive words are mapped onto upward and close-to-the-body locations and negative words are mapped onto downward and away-from-the-body locations; neutral words are placed midway. This way of placing words in relation to the body is consistent with an approach-avoidance effect such that “good” and “bad” things are kept close to and away from one’s body. We demonstrate for the very first time that this same pattern emerges when visual, auditory, tactile, and olfactory perceptual stimuli are placed on 3D physical space. We believe these results are significant in that the CC can be used as a new tool to diagnose emotion-related disorders.

## Introduction

The valence-space metaphor contends that stimuli’s valence is mapped onto physical space. In the case of the vertical plane, various studies have shown that positive and negative stimuli are associated with high and low locations, respectively (Meier and Robinson, 2004; Xie et al., 2014, 2015; Montoro et al., 2015; Sasaki et al., 2015). A handful of studies suggest that positive and negative items can be associated with rightward and leftward locations, respectively, when the task is performed by right-handed participants and the pattern reverses when the task is performed by left-handed participants (Casasanto, 2009; Casasanto and Chrysikou, 2011; Freddi et al., 2016). More recent evidence suggests, however, that no mapping occurs in this horizontal plane (e.g., Amorim and Pinheiro, 2018) and that factors such as age, gender, language, handedness, and, most importantly, valence exert no effect (Marmolejo-Ramos et al., 2017).

The stimuli used in those studies consisted of visual, verbal, or auditory stimuli (e.g., pictures, words, sounds) while at the same time being restricted to the mapping of one or at best two dimensions at the time (e.g., mapping of valence to vertical and/or horizontal space via computer screens or by paper-pencil tasks). In contrast, valence-to-space mapping using olfactory or tactile stimuli are virtually lacking, as are studies that target metaphorical mapping onto our physical reality of height, length, and width simultaneously. Recently, a study showed for the very first time how valenced concepts are placed onto 3D physical space. In that study, Marmolejo-Ramos et al. (2018) crafted a specific device (“the cognition cube”) that enabled allocating items in space such that *X*, *Y*, and *Z* Cartesian coordinates could be estimated. These authors not only confirmed the mapping of valenced concepts onto the vertical plane, but also disconfirmed the effects of various factors, including valence, on the horizontal plane. More importantly, their results showed that in the “depth” plane (*Z*-axis) positively- and negatively valenced concepts are placed closer to and away from the body, respectively. This result is line with an approach-avoidance effect (Solarz, 1960; Phaf et al., 2014; see also Meier et al., 2012; Topolinski et al., 2014; Godinho and Garrido, 2016).

Research on embodied cognition argues that concepts are built during the sensorimotor experience with the environment (Smith and Colunga, 2012; Martin, 2016). But concepts are more elaborate in that those perceptual and motor experiences are colored by the social (and, in turn, emotional) context in which experiences ensue (e.g., Marmolejo-Ramos et al., 2017a; see also Pecher, 2018). As to the specific case of perceptual experience, humans have built-in circuitry that enables processing such type of information via sensory systems (i.e., vision, audition, touch, smell, and taste) and the mental representation of the sensory input is known as percept (see Jepson and Richards, 1993). Thus percepts can be understood as a form of primitive concepts in that they are closer to sensory-to-perceptual than to social experiences^1^.

Despite percepts being primitive concepts, they do have associated valence. As recent studies indicate, tastes and shapes have associated valences such that, for example, sweet tastes and round shapes are associated with a positive valence (Velasco et al., 2014a,b, 2016). There is also evidence for associations between percepts and 2D space such that, for example, high-pitched sounds, which also have an associated positive valence, are mapped onto high spatial locations (Salgado-Montejo et al., 2016; but see Rusconi et al., 2006). By merging these results with those recently found by Marmolejo-Ramos et al. (2018), we hypothesized that percepts are placed in 3D space in the same fashion as are valenced concepts. That is, positive percepts will be placed in high locations (*Y*-axis) and near the participant’s body (*Z*-axis), negative percepts will be placed in low locations and far from the participant’s body, and neutral percepts will be placed in between these percepts regarding both planes. As to the *X*-axis, no consistent nor straightforward effect of valence, nor any other effect is expected. Perceptual stimuli from four sensory modalities are used and, as has been shown, some modalities dominate over others (see Colavita, 1974; Koppen and Spence, 2007; Rach et al., 2011). It is therefore expected that the modality of the percept – being the visual modality most likely to dominate overall – can lead to differences in the strength of associations between percepts and space.

## Materials and Methods

### Participants

Forty-four undergraduate and graduate students participated in the experiment (29 females; *Mdn*_age–females_ = 26 ± 4.44_MAD_, *range*_age–females_ = 18–52 years, 2 left-handed; *Mdn*_age–males_ = 25 ± 5.93_MAD_, *range*_age–males_ = 19–33 years, 1 left-handed). This sample size was suggested by a power analysis for a general linear model with five fixed predictors that, as a full model, explained at least 25% of the variance in the dependent variable under a 5% Type I error (α) and an 80% power. (For details regarding the power estimation, see Marmolejo-Ramos et al., 2018) None of the participants reported any known visual, tactile, olfactory, auditory, or related sensory impairment. Forty percent of the participants were non-native Swedish speakers who reported good-to-excellent command of the English language. All participants received course credit or cinema tickets or participated voluntarily. The study’s protocol was approved by the ethics committee of the Department of Psychology at Stockholm University (experiment code 67/16). All subjects gave written informed consent in accordance with the Declaration of Helsinki (World Medical Association [WMA], 2013).

### Stimuli and Materials

Nine highly familiar stimuli (3 positive, 3 neutral, and 3 negative) were selected for each of the sensory modalities vision, audition, touch, and smell (see Table 1). The images were selected from the IAPS data set (Lang et al., 2008; items’ codes: 1710, 1440, 1460, 7705, 7547, 7011, 1111, 7380, and 3019). The sounds were selected from a large sound database available on CDs (BBC Sound Effects Library – Original Series, United Kingdom) and from an online collaborative sound database (Freesound ^2^, item’s codes: bbc 74, bbc 19, bbc76, bbc 101, bbc 98, bbc 88, bbc 100, bbc 32, bbc 01). The sounds were edited to a duration of 3 s, an intensity of 50 dB, and converted to stereo by using a digital audio editor and recording program (Audacity; see Cornell Kärnekull et al., 2016). The textures and the smells were selected from previous work where valence ratings were available (Ekman et al., 1965; Etzi et al., 2014; Cornell Kärnekull et al., 2016). Although the valence of all stimuli was assessed in previous studies, their valence was corroborated in the present study via rating scales (*positive*: images = 9 ± 1.48, sounds = 8 ± 1.48, textures = 7.1 ± 2.14, smells = 7.55 ± 1.85; *neutral*: images = 5 ± 1.48, sounds = 5 ± 1.48, textures = 4.9 ± 2.22, smells = 5.05 ± 3.26; *negative*: images = 3 ± 2.96, sounds = 2 ± 2.96, textures = 4.95 ± 2.29, smells = 2.2 ± 2.29)^3^. A computer-based version of VAS (visual-analog scales) was implemented in PsychoPy (Peirce, 2007) for the olfactory and tactile stimuli, and a paper-pencil version was used for the visual and auditory stimuli^4^.

**Table 1:** Test stimuli across each sensory modality.

Valence	Sensory modality			
	Images	Sounds	Textures	Smells
	Dogs	Sea wash calm	Cotton	Cola
Positive	Seal	Horse trot	Satin	Peach
	Kitty	Turning book pages	Tinfoil	Banana
	Closet	Electric kettle	Tulle	Lavender
Neutral	Bridge	Cards shuffling	Oasis	Mushroom
	Gasoline	Pieces of glass	Kitchen sponge	Cut grass
	Snakes	Electronic drill	Abrasive sponge	Onion
Negative	Roaches	Burners and pilot light	Cardboard	Garlic
	Carcass	Bathroom fan	Sandpaper	Fish

**FIGURE 1:**
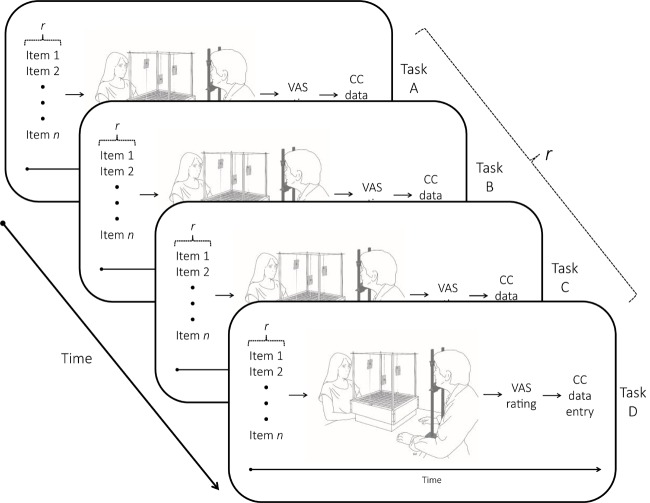
Illustration of the experimental sequence. Presentation of the four modalities (visual, auditory, tactile, olfactory) was counterbalanced, and the respective modality items (images, sounds, smells, textures for Tasks A ∼ D) were randomly presented in each modality set (*r*). The following fixed sequence of events occurred within each task: (i) stimuli were presented one by one (item 1… *n*); (ii) stimuli were allocated spatially within the cognition cube (CC); (iii) stimuli were rated (VAS rating); and (iv) the *X*, *Y*, and *Z* coordinates of each stimulus were measured and recorded.

The cognition cube was used to measure the allocation of the different sensory stimuli in 3D space (see Marmolejo-Ramos et al., 2018, including its Supplementary Material, for details of this new device). Each stimulus had a unique code name that was printed and placed within a small plastic badge with magnets that could be positioned onto a vertical metal rod (see Figure 1). In this way, the horizontal and depth location of a stimulus could be pinpointed by moving the metal rod perpendicularly to the floor of the cube while the vertical (*Y*) coordinate was given by the location of the badge on the rod itself.

### Procedure

All participants were tested individually and by the same experimenter. First, participants were provided with the following written instruction which also was orally presented by the experimenter.

“We are interested in knowing how people allocate sensory stimuli in space. In this task, this [pointing to the cube] will be your space. This space is confined within these eight edges [pointing to the internal corners of the cube] and is bounded by this farthest limit [pointing to the farthest limit in the *Z* plane], this closest limit [pointing to the section of the cube closest to the participant], this lowest limit [pointing to the floor of the cube], this highest limit [pointing to the ceiling of the cube], this rightmost limit [pointing to the rightmost internal edge of the cube] and this leftmost limit [pointing to the leftmost internal edge of the cube]. You will be presented with images, sounds, smells, and textures; each in separate blocks and item by item. Your task is to allocate each sensory stimulus anywhere within this cube [the experimenter made clear that any location within the space can be used] by pointing to where you would place it [the experimenter asked the participant to use his/her dominant hand]. Please make your location choice while looking at the cube [this happened while the person’s head rested on a chin rest]. Once you have decided where to place it, please point at the desired location. If you cannot reach the location, you can stand up and point at it. In order to translate your decision into the cube, please hold your hand in the spot you decided to place the stimulus [at this point the experimenter attached a badge with the item’s code name to a metallic rod]. After allocating all of the items in a modality task, you will be asked to rate the pleasantness of the stimuli. Then, the same procedure will be applied for the next modality.”

The participant sat in front of the cube with the chin rest at a distance (∼40 cm) that allowed him/her to perceive and reach all corners within the cube. This procedure ensured that the visual field of the participant could cover each area in the cube equally well, i.e., similar ocular movements for looking to rightward-leftward and upward-downward directions. Also, the experimenter reminded the participant that the goal of the study was to know how people put “objects” in space with no connotation of the valence of the stimuli. The participant was reminded he/she could choose any location within the cube and could do so by pointing to that location. By means of a chin rest, the participant’s eye level was aligned to the center of the cube. The experimenter always sat opposite to the participant (see Figure 1).

Spatial allocations were performed for the four sensory modalities (visual, auditory, touch, and olfactory) separately comprising a total of 36 stimuli. Modality order presentation was counterbalanced, and presentation of the items within each modality was randomized. For each modality item presented, the experimenter asked the participant to spatially allocate the stimulus in the cube. When the participant pointed to the selected location, he/she was asked to hold their hand in the selected position while the experimenter positioned the badge with the code name of the specific stimulus (see Table 1). The code names were printed in black ink on 8 cm (W) × 3 cm (L) white paper and placed in a transparent plastic badge. Once all modality items had been placed within the cube, the participant was asked to rate the valence of the stimuli presented. (Items were presented in random order). Finally, the experimenter recorded the *X*, *Y*, and *Z* coordinates for each allocated item before the next modality task was presented. The entire experimental session lasted between 45 and 60 min (see Figure 1). At the end of each test session, all participants were asked whether they were aware of the purpose of the study. None of the participants reported knowledge of the study’s aims.

### Design and Statistical Analyses

The dependent variables were the Cartesian 3D coordinates. The *X*, *Y*, and *Z* coordinates of each stimulus took integer values between −20 and 20. The subjective valence ratings of the stimuli took values between 0 (negative) and 10 (positive). The valence ratings were examined in relation to the location of the stimuli in each of the 3D coordinates.

The independent variables were the stimuli’s valence (positive, neutral, negative), sensory modality (visual, auditory, touch, and olfactory), the interaction between these two factors, participants, and stimuli. These last two factors were treated as random effects. The model with all main, interaction, and random effects was assessed via a robust linear mixed-effects model (here LMMr; implemented in the function “rlmer” in the R package “robustlmm;” Koller, 2016). The amount of variance explained by the full model (i.e., fixed and random effects) was estimated via the pseudo-*R^2^* for (generalized and linear) mixed-effect models with random intercepts (Nakagawa and Schielzeth, 2013 for an extension of this method to random slopes models, see Johnson, 2014,). This method is implemented in the function “r.squaredGLMM” in the “MuMIn” R package. Its output provides both the *R^2^* of the fixed-effects (here $R_{\text{f}}^{2}$) and the *R*^2^ of the full mixed-model (here $R_{\text{m}}^{2}$). These values are here reported as percentages. ANOVA results for the main effects and interactions were obtained via a rank-based test statistic (Brunner et al., 2017). (This test is implemented in the function “rankFD” in the “rankFD” R package).

Supplementary analyses were performed on the *X*, *Y*, and *Z*-values of each sensory modality in order to assess the effects of other covariates (see Supplementary Material). *Post hoc* pairwise comparisons of dependent measures were performed on 20% bootstrapped trimmed means (via the function “pairdepb” in the “WRS2” R package; see Wilcox, 2017). For these pairwise comparisons the average differences, 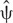, and 95% CIs around them are reported. (Significant differences are evidenced by the 95% CIs not containing the value of 0). The results are represented via 3D- and violin-plots (Hintze and Nelson, 1998).

Associations among locations in the *X*, *Y*, and *Z* coordinates and the items’ valence ratings were assessed via the maximal information coefficient [here *r*_MIC_; this criterion measures relationships ranging between 0 (no relationship) and 1 (noiseless functional relationships)], (Reshef et al., 2011) accompanied by the *p*-value of the percentage bend correlation; here *p*_pb_ (the function “mine” in the “minerva” R package performs the *r*_MIC,_ and the function “pbcor” in the “WRS2” R package performs the percentage bend correlation; Wilcox, 2017).

## Results

The effect of valence to space mapping was in accordance with our hypothesis. The analyses of the associations among locations in the three axes and the subjective valence ratings for the stimuli suggested that the more positively items were rated, the higher their location in vertical (Y) space (Images: *r*_MIC_ = 0.21, *p*_pb_ < 0.0001; Sounds: *r*_MIC_ = 0.13, *p*_pb_ = 1.79*e*^−7^; Smells: *r*_MIC_ = 0.13, *p*_pb_ < 0.001; Textures: *r*_MIC_ = 0.15, *p*_pb_ 2.71*e*^−6^). The more negatively items were rated, the further away from the body they were placed in the depth (Z) plane (Images: *r*_MIC_ = 0.10, *p*_pb_ = 0.002; Sounds: *r*_MIC_ = 0.12, *p*_pb_ = 4.66*e*^−5^; Smells: *r*_MIC_ = 0.14, *p*_pb_ = 0.015; Textures: *r*_MIC_ = 0.14, *p*_pb_ = 3.86*e*^−6^). In the horizontal (*X*) plane, no associations were reliable (all *p*_pb_’s > 0.08).

**FIGURE 2:**
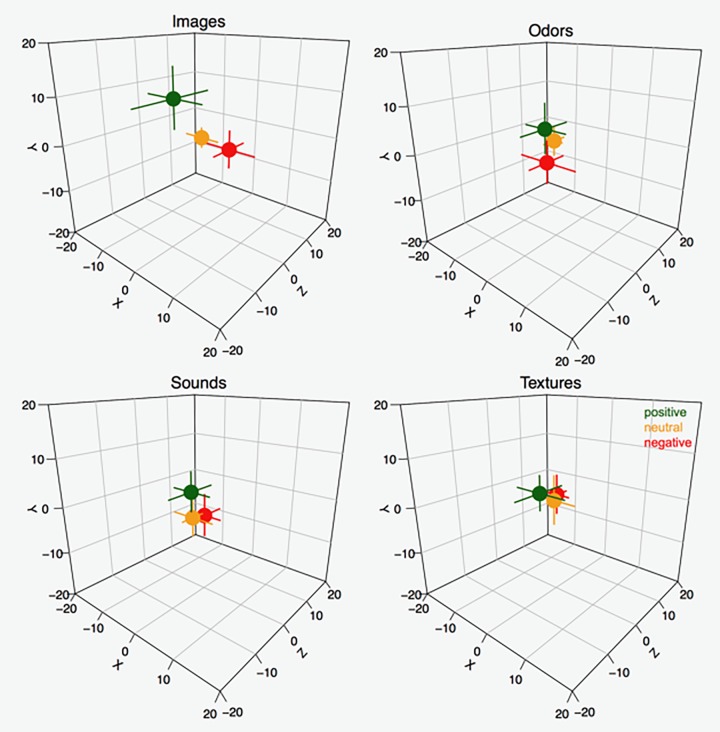
Median *X*, *Y*, and *Z* positions for positive, neutral, and negative stimuli in the four sensory modalities. In order to understand the spatial distribution of the data in relation to the participants’ perspective, assume the participants were facing the cube from the left angle; i.e., in front of the *X* axis. Error bars represent 95% CIs around the median (estimated as ±1.58 ⋅ $\left(\frac{\mathrm{IQR}}{\sqrt{n}}\right)$, where *IQR* = interquartile range and *n* = sample size).

**FIGURE 3:**
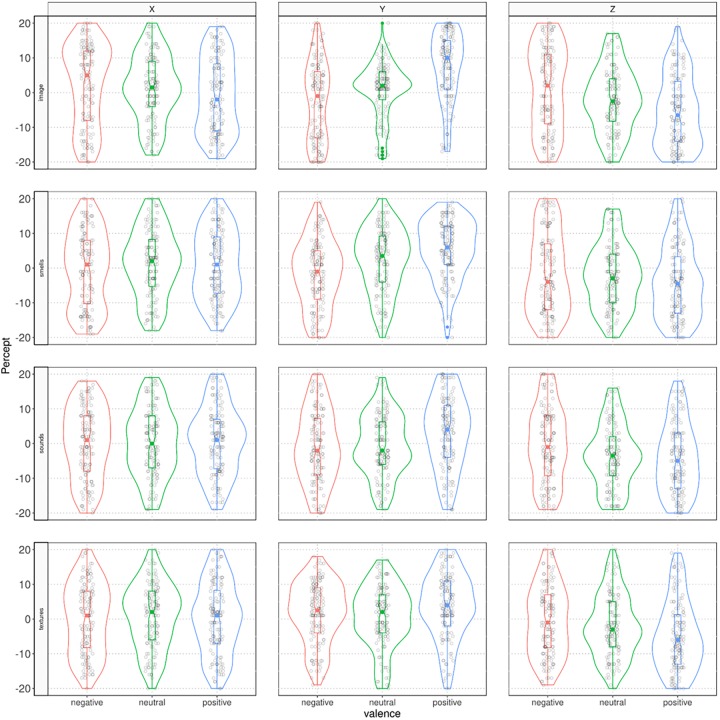
Violin-plots displaying the distribution of locations of valenced stimuli in each sensory modality in the *X*, *Y*, and *Z* spatial axes.

The results from the ANOVA indicated significant main effects of valence, *F*(1.99,1538.73) = 51.10, *p* < 0.00019 (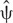
_negativevs.neutral_ = −3.42 [−9.33, 2.47], 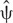
_negativevs.positive_ = −7.89 [−14.13, −1.65], and 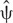
_neutralvs.positive_ = −4.46 [−9.68,.75]), and sensory modality, *F*(2.99, 1538.73) = 2.85, *p* = 3.59*e*^−2^. (The only significant pairwise differences were: 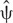
_smellsvs.images_ = −7.21 [−12.78, −1.64] and 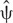
_smellsvs.textures_ = −8.32 [−14.91, −1.73]) in the Y axis, although no significant interaction effect between valence and sensory modality was observed [*F*(5.94, 1538.73) = 5.21, *p* = 2.67*e*^−5^]. Although the test suggested that in the Z axis there was an effect of valence, *F*(1.98,1538.50) = 21.14, *p* = 9.94*e*^−10^, *post hoc* comparisons did not support that claim in that the 95% CIs of the average differences in all pairwise comparisons contained 0. The effects of sensory modality *F*(2.99,1538.50) = 0.32, *p* = 0.80 and the interaction between valence and modality in the *Z* axis were also non-significant, *F*(5.93,1538.50) = 1.08, *p* = 0.37. Moreover, and in accordance with our hypothesis, the ANOVA-type results indicated that no main effect or interaction was evident in the *X* axis for either valence or modality [valence: *F*(1.99,1546.63) = 0.65, *p* = 0.52; sensory modality: *F*(2.99,1546.63) = 0.24, *p* = 0.86; and their interaction: *F*(5.95,1546.63) = 1.04, *p* = 0.39] (see Table 2 for results of the LMMr and Figure 2, 3).

**Table 2:** Results of the robust, linear mixed-effects models for each of the dependent variables.

DV		Factors			Random (intercept) effect (Var, *SD*)		%VE ($R_{\text{f}}^{2}$, $R_{\text{m}}^{2}$)
		Fixed main effect and interaction (estimate (SE) [*t*-value]				*P*	*I*	
X	V	n: −1.12 (1.40) [−80] ne: 0.68 (1.40) [0.49]	M	s: −0.12 (1.40) [−0.09] i: −1.62 (1.40) [−1.16] t: −0.61 (1.40) [−0.44]	V•M	n_ˆ_i: 4.68 (1.98) [2.36] ne_ˆ_i: 1.45 (1.98) [0.73] n_ˆ_s: 0.91 (1.98) [0.46] ne_ˆ_s: −0.96 (1.98) [−49] n_ˆ_t:1.12 (1.98) [0.57] ne_ˆ_t: 0.04 (1.98) [.02]	0,0	0,0	0.62, 0.62
Y	V	n: −7.72 (1.09) [−7.03] ne: −3.32 (1.09) [−3.02]	M	s: −2.35 (1.09) [−2.15] i: 2.43 (1.09) [2.21] t: −2.04 (1.09) [−1.86]	V•M	n_ˆ_i: −3.11 (1.55) [2] ne_ˆ_i: −3.92 (1.55) [−2.52] n_ˆ_s: 3.04 (1.55) [1.95] ne_ˆ_s: −0.84 (1.55) [−0.54] n_ˆ_t:6.07 (1.55) [3.91] ne_ˆ_t: 0.33 (1.55) [0.21]	11.68,3.41	0, 0	9.90,21.95
Z	V	n: 2.63 (1.30) [2.02] ne: 1.88 (1.30) [1.44]	M	s: 0.10 (1.30) [.07] i: −1.04 (1.30) [−0.8] t: −0.68 (1.30) [−0.52]	V•M	n_ˆ_i: 4.18(1.84) [2.27] ne_ˆ_i: 1.62 (1.84) [0.88] n_ˆ_s: 1.38 (1.84) [0.74] ne_ˆ_s: −1.16 (1.84) [−0.63] n_ˆ_t: 1.80 (1.84) [0.98] ne_ˆ_t: 1.16 (1.84) [0.63]	9.94,3.15	0,0	3.30,11.55

*V, valence [n, negative; ne, neutral (positive is the reference level)], M, sensory modality (s, sounds; i, images; t, textures (smells is the reference level)], VM, interaction between V and M (positive and smells are the reference levels), P, participants; I, items. Var, variance; SD, standard deviation; SE, standard error. DV, dependent variable. %VE: percentage of variance explained [$R_{f}^{2}$: fixed-effects model, $R_{m}^{2}$: mixed-effects model (fixed and random effects)].*

Supplementary analyses (see Supplementary Material) further indicate that the valence of the sensory stimuli is a major factor for predicting the allocation variation across the coordinates. In general, valence proved a stronger predictor than modality when allocating sensory stimuli in 3D space. Evidence of this is that the valence of the sensory modality was ranked as the most important variable in most cases across tasks and axes and exhibited the largest associated *t*-values (see Supplementary Table S1). The percentage of variance explained by all models considered suggests that the allocation of valenced modalities is more salient in the *Y* axis, followed by the *Z* and *X* axes. (See also results of the ANOVA above). Finally, the relationships among valenced modalities and space proved more salient for the visual stimuli, followed by auditory, tactile, and olfactory stimuli. (See also Figure 2, 3).

## Discussion and Conclusion

This study had the goal of examining the allocation of valenced percepts in four sensory modalities in 3D space. The results showed that positive percepts are placed in high locations, negative percepts are placed in low locations, and neutral percepts fall in between. Positive percepts are placed closer to the body, negative percepts are placed farther from the body, and neutral percepts fall in between. In the horizontal plane, the effect of a percept’s valence does not manifest. Overall, these results agree with those reported in a recent unique study in which valenced concepts were allocated in 3D space (see Marmolejo-Ramos et al., 2018).

The present study also showed that the effect of the percepts’ valence and their allocation in space was largest in the vertical plane (*Y* axis), followed by the “depth” and horizontal planes (*Z* and *X* axes, respectively). This result also chimes with the study of Marmolejo-Ramos et al. (2018), which is the first and only available study on the allocation of valenced concepts onto 3D space. Finding that covariates such as handedness, gender, age, language, and valence play no role in how valenced percepts are placed on the horizontal plane (see Supplementary Material) is also in line with studies which consistently show this same situation in the allocation of concepts in 2D (e.g., Marmolejo-Ramos et al., 2013, 2017) and, more recently, 3D (Marmolejo-Ramos et al., 2018) space (see Supplementary Material). In the current study, the null effect of valence and other factors in the *X* axis was manifest across all sensory modalities. Only in the case of valenced images, negative images tended to be placed rightward while positive images tended to be placed leftward. The non-significant associations between the items’ ratings and the *X* axis values provide further evidence against any link between percept’s valence and the horizontal plane in the sensory modalities studied.

Results in the *Z* plane are in line with an approach-avoidance paradigm such that positive percepts tended to be placed toward the body while negative percepts were placed away from the body. This pattern was evident across sensory modalities (except for the case of smells). In the vertical plane, positive percepts are placed in high locations while negative percepts are placed in low locations (except for the case of textures). The significant associations between the percepts’ valence ratings and the stimuli’s coordinates in the *Y* and *Z* axes, however, supports these claims. Differences in the allocation of percepts in each modality might be due to the dominance that some modalities have over others, as has been shown in the case of vision over olfaction (see Sakai et al., 2005; Sugiyama et al., 2006).

This study extends the results of Marmolejo-Ramos et al. (2018) to the case of percepts and, by the same token, validates the cognition cube as a suitable device for the study of valenced items and their association with 3D space. Specifically, the current study indicates that people map affective visual, auditory, olfactory and tactile information onto physical space in a systematic manner that reflects conceptual metaphors (see Lakoff and Johnson, 1980; Lakoff, 2014; for a recent proposal on this topic see Marmolejo-Ramos et al., 2017a). This study thus suggests that in normal adult samples, valenced concepts and percepts are placed onto 3D space differentially (as stated in the discussion). Future work is needed to investigate whether neurocognitive (e.g., alexithymia) and developmental factors (e.g., children) reflect in how concepts and percepts are allocated in space. Likewise, we believe the cognition cube could be used as a novel approach to diagnose emotion-related disorders (we are indeed working on this front and some preliminary results indicate this to be the case).

## Author Contributions

FM-R and AA designed the study. FM-R wrote the manuscript with input from AA, CT, and ML. CT administered the experiments. FM-R and RO carried out the data analyses.

## Conflict of Interest Statement

The authors declare that the research was conducted in the absence of any commercial or financial relationships that could be construed as a potential conflict of interest.

## Footnotes

1 It is assumed in the current study that while sensations are primitive experiences elicited by sensory stimulation, perceptions are meaningful experiences of objects or events (Mather, 2016, p. 26; see also Treuer, 2003). Thus the concept of percept used herein is closer to a perceptual than to a sensory experience. In line with this conjecture, it is further entertained that perceptual stimuli rather than sensory stimuli are the physical entities that elicit percepts (for some philosophical elaborations, see Prinz, 2006; for a recent reference to “percepts” in the context of embodied cognition research, see Miller et al., 2018).

2 www.freesound.org

3 A significant difference between “neutral” and “negative” textures could not be achieved. As has been shown in the case of images, it is sometimes difficult to rate some items as truly neutral (Schneider et al., 2016). The dispersion measures (*MAD*) associated to textures and smells seem greater than those of images and sounds. However, examining the ratio of the averaged coefficients of variation (such that *CV* = *MAD*/*Mdn*) for the two pairs within each of the three types of items showed ratios below 2. Also, while it is true the averaged *CV*s were larger for positive and neutral textures and smells than for positive and neutral images and sounds, this pattern reversed in the case of negative items {in R language: mean[c(CV.smells, CV.texures)] < mean[c(CV.images, CV.sounds)]}. Finally, although participants rated items’ valence, items’ arousal was not rated; this is a measurement that needs to be accounted for in future studies.

4 While ratings for images and sounds relied on a typical *n*-point Likert-type scale, the ratings for smells and textures used a visual analog-type scale. Therefore, while the data from the Likert-type scale consisted of a bounded discrete distribution (i.e., integer values between the minimum and maximum values of the scale), the data from the VAS-type scale resembled a bounded continuous distribution (i.e., values with decimal places ranging from the minimum to the maximum values of the scale).
