# Supplementary material for: The Allocation of Valenced Percepts Onto 3D Space
Source: Front Psychol. 2019 Feb 27;10:352. doi: 10.3389/fpsyg.2019.00352 (PMC6400994; doi:10.3389/fpsyg.2019.00352)
Supplement: Supplementary file 1 [file Table_1.DOCX]

**Supplementary material**

The data were analysed via linear regression (see Box, 1966; Schneider, Hommel, & Blettner, 2010; see also appendix A in Stauffer, 2008). The dependent variables were the values in X, Y, and Z coordinates of each of the sensory modalities and the independent variables were handedness (left- and right-handers; being ‘left’ the reference level), gender (males and females; being ‘males’ the reference level), valence (positive, neutral, and negative; being ‘positive’ the reference level), language (Swedish and non-Swedish; being ‘Swedish’ the reference level), age, participant, and items. These last two factors were treated as random-effects (specifically, as random intercepts) and all other factors were treated as fixed-effects in a robust linear mixed-effects model (here LMMr; implemented in the function ‘rlmer’ in the R package ‘robustlmm’; Koller, 2016). For reasons discussed elsewhere (Bates et al., 2015; see also ‘pvalues’ in the lme4 R package), *p*-values associated with the coefficient estimates are not provided in the output. Although such *p*-values could be approximated via a standard normal distribution when the sample size is relatively large (e.g. *n*>30), we use the *t*-value of two as a cut-off ^[[1]](#footnote-1)^.

The importance of the fixed factors in each model was further assessed based on the pattern of results given by robust least angle regression (Khan, van Aelst, & Zamar, 2007; Zhang & Zamar, 2014; function ‘rlars’ in the R package ‘robustHD’), all-subsets regression (Miller, 1984; function ‘leaps’ in the R package ‘leaps’), and relative importance (Grömping, 2015; implemented in the R package ‘relaimpo’, Grömping, 2006) analyses.

The amount of variance explained by the full model (i.e. fixed and random effects) was estimated via the pseudo-*R^2^* for (generalized and linear) mixed-effect models with random intercepts (Nakagawa & Schielzeth; 2013; see Johnson, 2014, for an extension of this method to random slopes models). This method is implemented in the function ‘r.squaredGLMM’ in the ‘MuMIn’ R package and its output provides both the *R^2^* of the fixed-effects (here $R_{f}^{2}$) and the *R*^2^ of the full mixed-model (here $R_{m}^{2}$). These values are here reported as percentages.

**Results**

Table S1 shows the results of the models. As stated in the manuscript, these analyses indicate that the valence of the sensory stimuli is a major factor for predicting the allocation variation across the coordinates. In general, valence proved a stronger predictor than modality when allocating sensory stimuli in 3D space. Evidence of this is that the valence of the sensory modality was ranked as the most important variable in most cases across tasks and axes and exhibited the largest associated *t*-values (see Table S1). The percentage of variance explained by all models considered suggests that the allocation of valenced modalities is more salient in the Y axis, followed by the Z and X axes (see also results of the ANOVA reported in the manuscript). Finally, the relationships among valenced modalities and space proved more salient for the visual stimuli, followed by auditory, tactile, and olfactory stimuli (see Figures 2 and 3 in the manuscript).

*Table S1*. Results of the robust linear mixed-effects models for each of the dependent variables in each task. Variables with *t*-values larger than two are shaded. Im: allocation of images; So: allocation of sounds; Te: allocation of textures; Sm: allocation of smells. V: valence (n: negative; ne: neutral), G: gender (f: female), L: language (nS: non-Swedish), H: handedness (r: right-hander), A: age, P: participants, I: items. Var: variance, SD: standard deviation, SE: standard error. DV: dependent variable. %VE: percentage of variance explained ($R_{f}^{2}$*:* fixed-effects model, $R_{m}^{2}$: mixed-effects model [fixed and random effects]).

| **Task** | **DV** | **Factors** | | | | | | | | | | | **%VE**  **(**$R_{f}^{2}$***,***  $R_{m}^{2})$ |
| --- | --- | --- | --- | --- | --- | --- | --- | --- | --- | --- | --- | --- | --- |
|  |  | **Fixed effects (estimate (SE) [*t*-value]) ^ϒ^** | | | | | | | | | **Random (intercept) effects**  **(Var,**  **SD)** | |  |
|  |  |  | | | | | | | | | **P** | **I** |  |
| **Im** | **X** | V | n:  3.36 (1.48) [2.26] | G | f:  1.35 (1.34) [1.01] | L | nS:  .65 (1.28) [.50] | H | r:  -.92 (2.47) [-.37] | A:  .0 (.07) [.02] | 0,  0 | 0,  0 | 1.74,  1.74 |
|  |  |  | ne:  2.05 (1.48) [1.37] |  |  |  |  |  |  |  |  |  |  |
|  | **Y** | V | n:  -10.66 (1.15) [-9.24] | G | f:  2.03 (1.35) [1.50] | L | nS:  1.96 (1.30) [1.50] | A:  .09 (.07) [1.22] | H | r:  2.45 (2.50) [.97] | 6.57,  2.56 | 0,  0 | 20.30,  26.12 |
|  |  |  | ne:  -7.07 (1.15) [-6.13] |  |  |  |  |  |  |  |  |  |  |
|  | **Z** | V | n:  6.82 (1.45) [4.68] | G | f:  -1.66 (1.31) [-1.26] | L |  | H | r:  -1.37 (2.42) [-.56] | A:  .04 (.07) [.52] | 0,  0 | 0,  0 | 6.23,  6.23 |
|  |  |  | ne:  3.44 (1.45) [2.36] |  |  |  |  |  |  |  |  |  |  |
| **So** | **X** | L | nS:  1.50 (1.16) [1.29] | G | f:  1.07 (1.20) [.89] | V | n:  -.14 (1.35) [-.11] | H | r:  -.52 (2.25) [-.23] | A:  -.0 (.07) [-.02] | 0,  0 | 0,  0 | .69,  .69 |
|  |  |  |  |  |  |  | ne:  -.27 (1.35) [-.20] |  |  |  |  |  |  |
|  | **Y** | V | n:  -4.77 (1.09) [-4.34] | L | nS:  1.91 (1.84) [1.03] | H | r:  2.14 (3.57) [.6] | G | f:  1.40 (1.90) [.73] | A:  -.02 (.11) [-.22] | 23.63,  4.86 | 0,  0 | 6.18,  28.47 |
|  |  |  | ne:  -4.31 (1.09) [-3.92] |  |  |  |  |  |  |  |  |  |  |
|  | **Z** | V | n:  3.88 (1.35) [2.86] | L | nS:  -.46 (1.16) [-.39] | G | f:  -.59 (1.20) [-.49] | H | r:  -.41 (2.25) [-.18] | A:  -.0 (.07) [-.01] | 0,  0 | 0,  0 | 2.48,  2.48 |
|  |  |  | ne:  .84 (1.35) [.61] |  |  |  |  |  |  |  |  |  |  |
| **Te** | **X** | L | nS:  2.02 (1.17) [1.72] | H | r:  2.98 (2.25) [1.32] | A:  -.03 (.07) [-.45] | G | f:  1.13 (1.26) [.90] | V | n:  .04 (1.34) [.03] | 0,  0 | 0,  0 | 1.90,  1.90 |
|  |  |  |  |  |  |  |  |  |  | ne:  .68 (1.34) [.50] |  |  |  |
|  | **Y** | L | nS:  2.60 (1.55) [1.67] | V | n:  -1.72 (1.93) [-.89] | G | f:  1.86 (1.67) [1.11] | H | r:  -1.69 (2.99) [-.56] | A:  .0 (.09) [.07] | 16.16,  4.02 | 4.07,  2.01 | 4.13,  29.72 |
|  |  |  |  |  | ne:  -2.81 (1.93) [-1.45] |  |  |  |  |  |  |  |  |
|  | **Z** | V | n:  4.49 (1.12) [4.01] | H | r:  -6.04 (3.34) [-1.80] | A:  .01 (.10) [.16] | G | f:  1.25 (1.87) [.66] | L | nS:  1.27 (1.74) [.73] | 19.13,  4.37 | 0,  0 | 5.83,  24.19 |
|  |  |  | ne:  3.09 (1.12) [2.75] |  |  |  |  |  |  |  |  |  |  |
| **Sm** | **X** | V | n:  -1.18 (1.42) [-.83] | G | f:  -1.32 (1.34) [-.98] | L | nS:  .59 (1.22) [.48] | A:  -.04 (.07) [-.67] | H | r:  .92 (2.36) [.39] | 0,  0 | 0,  0 | 1.09,  1.09 |
|  |  |  | ne:  .62 (1.42) [.43] |  |  |  |  |  |  |  |  |  |  |
|  | **Y** | V | n:  -7.52 (1.62) [-4.63] | H | r:  -2.36 (2) [-1.18] | G | f:  .66 (1.13) [.59] | A:  .0 (.06) [.13] | L | nS:  -.07 (1.03) [-.07] | 0,  0 | 1.71,  1.30 | 9.79,  11.46 |
|  |  |  | ne:  -3.23 (1.62) [-1.99] |  |  |  |  |  |  |  |  |  |  |
|  | **Z** | H | r:  -5.59 (2.29) [-2.43] | V | n:  2.69 (1.37) [1.95] | A:  .12 (.07) [1.68] | L | nS:  1.36 (1.19) [1.14] | G | f:  -.62 (1.30) [-.47] | 0,  0 | 0,  0 | 3.57,  3.57 |
|  |  |  |  |  | ne:  1.97 (1.37) [1.43] |  |  |  |  |  |  |  |  |
| **Note.** ^ϒ^ Variables ranked (from left to right) from the most to the least important as suggested by robust least angle regression, all-subsets regression, and relative importance analyses. | | | | | | | | | | | | | |

**References**

Bates, D., Maechler, M., Bolker, B., & Walker, S. (2015). Fitting Linear Mixed-Effects Models Using lme4. *Journal of Statistical Software, 67*(1), doi:10.18637/jss.v067.i01.

Box, G. E. P. (1966). Use and abuse of regression. *Technometrics, 8* (4), 625-629.

Grömping, U. (2006). Relative importance for linear regression in R: the package relaimpo. *Journal of Statistical Software, 17* (1). DOI: 10.18637/jss.v017.i01

Grömping, U. (2015). Variable importance in regression models. *WIREs Computational Statistics, 7* (2), 137-152.

Johnson, P.C.D. (2014). Extension of Nakagawa & Schielzeth’s R^2^ _GLMM_ to random slopes models. *Methods in Ecology and Evolution, 5* (9), 944-946.

Khan, J. A., van Aelst, S., & Zamar, R. H. (2007). Robust linear model selection based on least angle regression. *Journal of the American Statistical Association, 102* (480), 1289-1299.

Koller, M. (2016). Robustlmm: an R package for robust estimation of linear mixed-effects models. *Journal of Statistical Software, 76* (6). DOI:10.18637/jss.v075.i06

Miller, A. J. (1984). Selection of subsets of regression variables. *Journal of the Royal Statistical Society. Series A (General), 147* (3), 389-425.

Nakagawa, S., & Schielzeth, H. (2013). A general and simple method for obtaining R^2^ from Generalized Linear Mixed-effects Models. *Methods in Ecology and Evolution*, *4* (2), 133–142.

Schneider, A., Hommel, G., & Blettner, M. (2010). Linear regression analysis – part 14 of a series on evaluation of scientific publications. *Deutsches Ärzteblatt International, 107* (44), 776-782.

Stauffer, H. B. (2008). *Contemporary Bayesian and frequentists statistical research methods for natural resource scientists.* N.J.: John Wiley & Sons.

Zhang, H., & Zamar, R. H. (2014). Least angle regression for model selection. *WIREs Computational Statistics, 6* (2), 116-123.

1. For example, the *p*-value of a *t*-value of 2 is ~. 022; in R terms: pnorm(2, lower.tail = FALSE). [↑](#footnote-ref-1)
